# Traditional Asian Herbs in Skin Whitening: The Current Development and Limitations

**DOI:** 10.3389/fphar.2020.00982

**Published:** 2020-07-07

**Authors:** Yibo Hu, Hongliang Zeng, Jinhua Huang, Ling Jiang, Jing Chen, Qinghai Zeng

**Affiliations:** ^1^ Department of Dermatology, Third Xiangya Hospital, Central South University, Changsha, China; ^2^ Institute of Chinese Materia Medica, Hunan Academy of Chinese Medicine, Changsha, China

**Keywords:** skin whitening, Asian herbs, traditional herbs, pigmentation, melanogenesis

## Abstract

In Asia, the market for whitening cosmetics is expanding rapidly, more and more people prefer to use natural products. Driven by natural product demand and technical advances, herbal research is also developing fast. Lots of studies reported that Asian herbal reagents can reduce melanogenesis, these findings provide evidence for the whitening application of Asian herbs. However, the current development status and challenges of herbal research need attention too. By reviewing these studies, different problems in studying herbal formulas, extracts, and active ingredients were presented. One of the most influential troubles is that the components of herbs are too complex to obtain reliable results. Thus, an understanding of the overall quality of herbal research is necessary. Further, 90 most cited Asian herbal studies on whitening were collected, which were conducted between 2017 and 2020, then statistical analysis was carried out. This work provided a comprehensive understanding of Asian herbal research in skin whitening, including the overall status and quality, as well as the focuses and limitations of these studies. By proactively confronting and analyzing these issues, it is suggested that the focus of herbal medicine research needs to shift from quantity to quality, and the new stage of development should emphasize transformation from research findings to whitening products.

## Introduction

Due to economic development and aesthetic needs, the global cosmetics market is unprecedentedly prosperous at present; likewise, the variety of cosmetics has also increased (Lee et al., 2016; Peltzer et al., 2016). Traditional herb-based natural products are putting into practical use as a new type of cosmetics, especially in skin whitening (Kanlayavattanakul and Lourith, 2018). Meanwhile, studies have screened abundant components from traditional herbs, most of them show favorable effects on pigmentation reduction. These findings have developed several hot products, such as arbutin and kojic acid (Leyden et al., 2011). In Asia, the application of traditional herbs in skin whitening has gotten more attention, two outstanding factors may contribute to this situation. One of them is local culture and aesthetic manner, generally, most Asians prefer white skin; the other factor is that Asia has a long history of using herbal medicine (Scarpa and Guerci, 1987; Gao et al., 2018). Driven by the booming whitening market in Asia, many studies focus on the effects of traditional herbs; meanwhile, Asian consumers show strong need and trust for herbal reagents (Kanlayavattanakul and Lourith, 2018).

However, reliable herbal reagents are still in short supply. Though studies provide a basic guarantee for whitening effects and safety of many Asian herbs, there are still a lot of issues to be addressed before they transformed into products. The problems come from several aspects, for example, many natural ingredients are unstable and only show mild effects, the limitations of research methods are also a tough issue (Bent and Ko, 2004; Efferth, 2017; Espinosa-Leal and Garcia-Lara, 2019). It’s not just that, we need to know the potential obstacles to the application of Asian herbs in skin whitening. Thus, this review provided a comprehensive understanding of the development status and problems of herbal research. In addition to summarizing the current findings, this work mainly focused on finding problems, which might help promote the application of traditional Asian herbs.

## The Increase of Asian Cosmetics Markets and Skin Whitening Demand

The Asian cosmetics market is growing rapidly. Immediately behind Europe and the United States, China, Japan, India, and Korea are major consumers of cosmetics. In China, the total retail sales of cosmetics exceeded 40 billion dollars over the past three years and still maintained a high growth rate according to the National Bureau of Statistics (NBS) (China, 2018). These statistics are impressive enough, it suggests that the Asian cosmetics market is unprecedentedly prosperous. In Asia, the whitening product is an important part of cosmetics, besides, natural products have great potential in the whitening market. From the perspective of consumers, natural products are more skin-friendly, so herbal reagents are readily accepted (Kanlayavattanakul and Lourith, 2018). Meanwhile, increased skin whitening demand leads to the explosion of herbal research.

## Regulation of Melanogenesis

Skin whitening is affected by many factors, but the whitening effect of most herbs depends on regulating melanin synthesis. As an important pigment, melanin is widely distributed in mucosa, retina, and ovary (Slominski et al., 2004), but it mainly deposits in the skin and plays a role in resisting ultraviolet radiation (UVR) (Pinkert and Zeuss, 2018). Melanin is a kind of indole derivative of 3,4 di-hydroxy-phenylalanine (DOPA) produced by melanocytes. It derives from tyrosine through a series of oxidative reactions in melanosomes (Sealy et al., 1982). The first step is known as the Raper-Mason pathway, which depends on tyrosinase (TYR) (Miranda et al., 1988), the key rate-limiting enzyme. Besides, several proteins are involved in the maturation of melanosomes, such as tyrosinase-related protein 1 (TYRP1) and dopachrome tautomerase (DCT or TYRP2) (Bertolotto et al., 1998). After that, melanosomes will be transported to nearby keratinocytes and deposit around the nucleus, where they work and eventually degrade. Proteins involved in this step are Ras-Related Protein Rab-27A (RAB27A), Myosin VA (MYO5A), Fascin Actin-Bundling Protein 1 (FSCN1) (Slominski et al., 2004), and so on. The complete process is called melanogenesis.

The regulation of melanogenesis is complex and can be divided into three aspects: melanin synthesis, transport, and degradation. Melanin synthesis is the most studied area, while transport and degradation are not well understood. First of all, the expression and activation of TYR have the most immediate impact on melanin synthesis and determines the color of human skin (Pavan and Sturm, 2019); second, oxidative stress is another vital factor in promoting melanin synthesis, though it also causes cell damage (Schalka, 2017). Moreover, Microphthalmia-associated transcription factor (MITF) is an important transcription factor that can upregulate the expression of TYR, TYRP1, and TYRP2. It’s known that several signaling pathways can regulate MITF, such as the MAPKs (ERK, JNK, and p38) signaling pathway (Kim et al., 2017; Xu et al., 2018); the canonical Wnt signaling pathway, and the cAMP/PKA/CREB signaling pathway (Wang et al., 2017b; Yun et al., 2018). Besides, neighboring keratinocytes and fibroblasts have great impacts (Joly-Tonetti et al., 2018; Koike et al., 2018). It is partly due to the effects of endocrine and paracrine cytokines secreted by keratinocytes and fibroblasts, such as alpha-melanocyte-stimulating hormone (α-MSH), stem cell factor (SCF) and endothelin1 (ET1) (Pei et al., 2018; Yuan and Jin, 2018). The regulating system also plays a role in hyperpigmentation diseases, such as freckles, chloasma, and sunburn (Slominski et al., 2004; Sulem et al., 2007). Most whitening cosmetics work *via* part of the regulating system. For example, ascorbic acid (AA) is a famous antioxidant, arbutin and kojic acid can inhibit tyrosinase activity (Seo et al., 2012; Qu et al., 2018). As reported, the mechanisms of other natural reagents are similar to these cosmetics, the details will be shown later.

## The Effects and Problems of Traditional Asian Herbs in Skin Whitening

The application of herbs in skin whitening starts quite early in Asia. The book *Shen Nong’s Herbal Classic* written in more than 2,000 years suggested that semen platycladi, the seed kernel of *Platycladus orientalis (L.) Franco*, can improve people’s complexion and appearance. The book *Theory of Medicine Nature* recorded that the rhizome of *Atractylodes macrocephala Koidz.* can ameliorate dark skin (Zhen, 2006). After thousands of years’ attempts in developing whitening reagents, lots of useful Asian traditional herbs have been recorded, and some of them have been studied in recent years (Xie and Yu, 1996). Generally, how traditional herbs are used can be divided into three types: formula (consisted of several herbs); extract (a mixture of several components, or a class of compounds, from the same herb), and active ingredient (a purified compound that has a definite molecular structure).

### Herbal Formulas

Herbal formulas work *via* the synergy of all components, each herb is necessary, the composition of formulas will follow a particular principle to increase efficacy and reduce side effects (Zhang, 1994). Herbal formulas account for a large proportion of herb use, but the formula-based study in whitening is rare. In Ye’s study, researchers screened 50 herbal reagents (32 herbs and 18 herbal formulas) and successfully identified three useful tyrosinase inhibitors: Qian-wang-hong-bai-san, Qiong-yu-gao, and San-bai-tang (Ye et al., 2010a). The authors further revealed that Qian-wang-hong-bai-san could inhibit the p38 MAPK and PKA signaling pathway, and San-bai-tang could inhibit the p38 MAPK signaling pathway (Ye et al., 2010b; Tsang et al., 2012). This formula exists for a long time, but it is the first time to reveal the mechanisms. In addition, a Thai herbal formula AVS073 was reported to affect melanogenesis *via* suppressing the activity of tyrosinase, as well as neutralize ROS *via* increasing glutathione (GSH) biosynthesis and glutathione S-transferase (GST) activity (Panich et al., 2013). A Korean formula LASAP-C exhibited anti-melanogenic efficacy *via* inhibiting melanogenic proteins (TYR, TYRP1, and TYRP2) both in cells and zebrafish (Kim et al., 2016). Moreover, India also has a long history of using herbal formulas and an ancient medical systems: Ayurveda. Therefore, many Indian formulas have been studied in recent years. Ubtan, a traditional formula, was reported to have anti-tyrosinase and antioxidant effects (Biswas et al., 2016). (The composition of formulas showed in **Table 1**.)

**Table 1:** The information of Asian herbal formulas.

Study	Formula	Species, concentration	Mechanisms	Models
Ye et al. (2010a); Tsang et al. (2012)	Qian-wang-hong-bai-san	tubers of *Bletilla striata* (Thunb.) Reichb. f., tubers of ***Sauromatum giganteum* (Engl.) Cusimano & Hett. (syn**. *Typhonium giganteum* Engl.**)**, pericarps of *Punica granatum* L., fruits of *Benincasa hispida* (Thunb.) Cogn., With the ratio of 1:1:1:1.	suppressing tyrosinase activity and expression; inhibiting p38 MAPK signaling pathway; inhibiting PKA/CREB signaling pathway	mushroom tyrosinase; B16 cells
Ye et al. (2010a)	Qiong-yu-gao	roots and rhizomes of *Panax* *ginseng* C. A. Mey., roots of *Rehmannia glutinosa* Libosch., rhizomes of *Smilax glabra* Roxb. With the ratio of 1:1:1.	suppressing tyrosinase activity	mushroom tyrosinase; B16 cells
Ye et al. (2010a); Ye et al. (2010b)	San-bai-tang	rhizomes of *Atractylodes macrocephala* Koidz., rhizomes of *Smilax glabra* Roxb., roots of *Paeonia lactiflora* Pall., With the ratio of 1:1:1.	suppressing tyrosinase activity and expression; inhibiting p38 MAPK signaling pathway and MITF expression	mushroom tyrosinase; B16 cells
Panich et al. (2013)	Ayurved Siriraj Brand Wattana formula (AVS073)	*Piper nigrum* (L.), *Boesenbergia rotunda* (L.) Manf., *Cyperus rotundus* (L.), *Tinospora crispa* **(L.) Hook.f. & Thomson**, *Terminalia chebula* Retz., *Cladogynos orientalis* **Zipp. ex Span** **.**, *Derris scandens* (Roxb.) Benth., *Anamirta cocculus* (L.) **Wight & Arn.**, ***Putranjiva roxburghii*** **Wall. (syn.** *Drypetes roxburghii* (Wall.)**)**, *Cinnamomum siamense* Craib., ***Ferulaa** assa-foetida* L., *Aegle marmelos* (L.) **Corrêa**, ***Conioselinum vaginatum* (Spreng.) Thell. (syn.** *Conioselinum univittatum* Turcz. **ex Kar. & Kir.)**, ***Aucklandia costus*** **Falc. (syn.** *Saussurea lappa* **(Decne.) Sch.Bip.)**, ***Cryptolepis dubia* (Burm.f.) M.R.Almeida (syn.** *Cryptolepis buchananii* Roem. & Schult.). Concentration not provided.	suppressing tyrosinase activity and mRNA; inhibiting ROS formation	G361 cells
Kim et al. (2016)	LASAP-C	root of *Rehmannia glutinosa* Libosch. var. purpurea Makino, 100g fruit of *Lycium chinense* Mill., 50 g root of *Scutellaria baicalensis* Georgi, 50 g root of *Angelica dahurica* (Hoffm.) Benth. & Hook.f. **ex Franch. & Sav.** 35 g	suppressing tyrosinase activity and expression	B16F10 cells zebrafish
Biswas et al. (2016)	Ubtan (UF-1 to UF-4)	rhizome of *Curcuma longa* L. seeds of *Cicer arietinum* L. heartwood of *Santalum album* L. With the ratio of 2:2:2 (UF-1), 1:2:2 (UF-2), 2:1:2 (UF-3) and 2:2:1 (UF-4)	antioxidant; sun protection; suppressing tyrosinase activity	*in vitro*: mushroom tyrosinase

These studies provide evidence for further research. However, some problems should be noted. First, the formulas processing, such as water decocting, is mainly based on ancient records or personal experience, but not a uniform standard. Apparently, differences in processing will affect the final composition (Bent and Ko, 2004). Moreover, the composition of formulas is incredibly complex (Yu et al., 2019), it brings uncontrollable interference to research. The inadequate knowledge of herbal formulas is a thorny issue for scientists under current conditions (Dai et al., 2019).

### Herbal Extracts

Due to the development of extraction techniques such as High Performance Liquid Chromatography (HPLC) and Ultra Performance Liquid Chromatography (UPLC), the components of herbal extracts can be identified now (Wang et al., 2017a). These techniques help separate crude herbal extracts into several classes: saccharides, glycosides, phenylpropanoids, quinones, flavonoids, terpenes, triterpenes, steroids, and alkaloids (Li et al., 2019). No doubt it is conducive to further research. Different from formulas, there are a lot of research reports on herbal extracts. For example, ginseng (*Panax ginseng* C.A.Mey.) leaves extract is found to be effective in moisturizing, anti-aging, freckle-removing, and skin whitening (Jimenez-Perez et al., 2018). Ganoderma lucidum polysaccharides can reduce melanogenesis by inhibiting cAMP/PKA and ROS/MAPK signaling pathways, as well as inhibiting paracrine effects (Hu et al., 2019; Jiang et al., 2019). Goji berry (*Lycium chinense* Mill.) root extract can result in depigmentation *via* suppressing oxidation, MAPK and PKA signaling pathways (Huang et al., 2014). *Gastrodia elata* Blume and *Foeniculum vulgare* **Mill**. fruits extracts can resist α-MSH or UV-induced melanogenesis (Nam and Lee, 2016; Shim et al., 2017). Essential oils from the leaves of *Pogostemon plectrantoides* Desf. were tyrosinase inhibitors (Suganya et al., 2015). What is more, a clinical study reported that polypodium leucotomos extract treatment is safe and effective for melasma patients (Goh et al., 2018).

As we all know, growing conditions (soil, water, climate), growth time, and harvest time have great impacts on herbs in cultivation (Yuan et al., 2016; Olennikov et al., 2017; Zhang et al., 2017). These factors will subsequently influence the components of herbal extracts, so do their effects (Bent and Ko, 2004). Furthermore, different extraction methods yield different ingredients (Lin et al., 2019). For instance, Wang et al. compared the effects of water and ethanol extracts of *Cuscuta chinensis* **Lam.** seeds in B16F10 cells and zebrafish; it is impressive that water extract inhibited tyrosinase activity, but ethanol extract worked oppositely (Wang et al., 2014). What is more, many ingredients widely exist in herbs, thus different herbal extracts may have similar components (Yuan et al., 2012; Wu et al., 2018), while the content of special ingredients tends to be lower (Ho et al., 2013; Tian et al., 2019). For these reasons, it is hard to guarantee the reliability of experimental results.

### Active Ingredients

With the renovation of extraction techniques, such as Enzyme-Assisted Extraction (EAE), Supercritical-Fluid Extraction (SFE), and Microwave-Assisted Extraction (MAE) (Bilal and Iqbal, 2020), a large number of active ingredients have been purified and identified, some of them show good performance in anti-tumor, anti-inflammatory, antioxidation, and skin whitening (Gao et al., 2019; Zeng et al., 2019b). Many famous herbs have been well studied, such as licorice (*Glycyrrhiza* uralensis **Fisch. ex DC.**), ginseng, and aloe (*Aloe vera* (L.) Burm.f.). Chen et al. confirmed that glabridin (extracted from licorice) reversibly inhibits tyrosinase in a non-competitive manner (Chen et al., 2016). Besides, floralginsenoside A (extracted from ginseng) showed anti-melanogenesis effects in cells and zebrafish *via* regulating MITF expression and ERK activation (Lee et al., 2017). Moreover, aloin (extracted from aloe) led to skin lightening *via* alpha-adrenergic receptor stimulation (Ali et al., 2012). Betulinic acid (extracted from *Dillenia indica* L.) exhibited non-competitive mode of tyrosinase inhibition (Biswas et al., 2017). Bixin and norbixin (from *Bixa orellana* L.) inhibited both melanin synthesis and tyrosinase activity (Anantharaman et al., 2016). 2-hydroxy-4-mehoxybenzaldehyde (MBALD) and its crude extract (extracted from *Hemidesmus indicus* [L.] R. Br. ex Schult.) could inhibit monophenolase activity (Kundu and Mitra, 2014). As the research object is more clear, these findings are more convincing to support the whitening effect of herbs.

Screening active ingredients is an important part of the herbal study, and it has great application prospects. But unlike the success in research, there are still many problems with herbal ingredients before they can be used. First, most natural ingredients are difficult to extract and purify on a large scale (Bai et al., 2014), and extraction costs are a key factor limiting the conversion of ingredients into products. Second, many herbal ingredients only show moderate effects and are unstable under normal conditions, their performance depends on the structure and properties (Manda et al., 2014; Ho et al., 2016; Lyles et al., 2017). Thus, the molecular structure of some ingredients will be further improved before use, research in this area is under development.

## The Status of Asian Herbal Research in Skin Whitening

In addition to finding problems by reviewing previous studies, it is also important to have a comprehensive understanding by statistics. Thus, we collected Asian herbal studies published between January 2017 and May 2020 and conducted a multifaceted analysis, aiming to understand the research status of Asian herbs in skin whitening, as well as to assess the overall quality and value of these studies. We used “skin whitening” and/or “Asian herb” as keywords to retrieve articles by Web of Science, the selected databases were Web of Science Core Collection, BIOSIS Citation Index, and MEDLINE^®^, the time frame was from January 2017 to May 2020. After that, we sorted the lists by academic citations and reviewed the 300 most cited articles in detail, then removed studies that were not associated with Asian herbs. Finally, we collected 90 studies for subsequent analysis. (The information of studies we collected were provided in *Supplementary Materials*.)

### Overview of Asian Herbal Research

**Figure 1:**
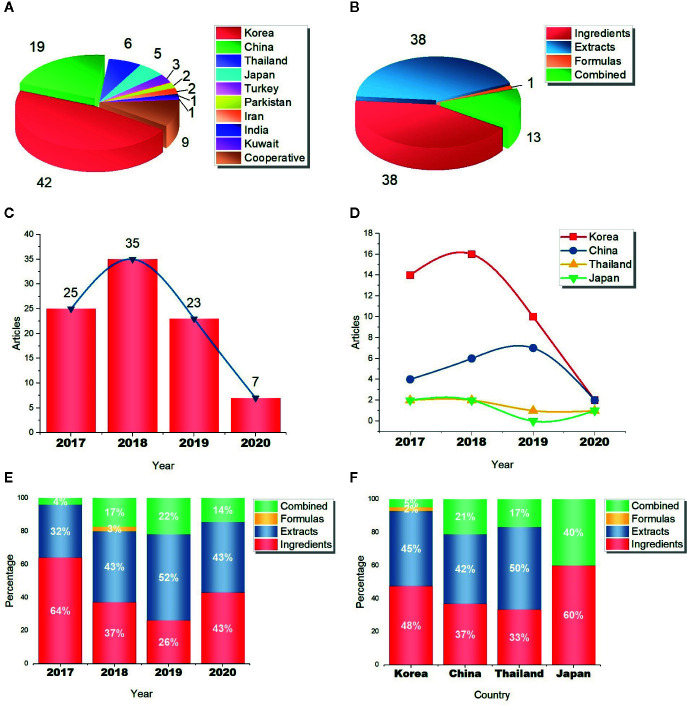
Overview of 90 Asian herbal studies in skin whitening. **(A)** The number of studies in different countries; **(B)** the number of studies on different reagent types; **(C)** the number of studies from 2017 to 2019; **(D)** the studies in four countries from 2017 to 2019; **(E)** the percentage of different reagent types from 2017 to 2019; **(F)** the percentage of different reagent types in four countries.

Similar to the size of cosmetics markets, 72 studies were conducted in South Korea (42 studies, 46.7%), China (19 studies, 21.1%), Thailand (6 studies, 6.7%), and Japan (5 studies, 5.6%); 9 studies were conducted in Turkey, Pakistan, Iran, India, and Kuwait; the other 9 studies were cooperative projects between several Asian countries (**Figure 1A**). The geographical distribution of studies is related to the scientific research level, to some extent, it also can reflect the cosmetic market size and whitening demand. In another perspective, though only a few studies were conducted in countries other than Korea, China, Thailand, and Japan, the optimistic explanation is that the whitening markets in these countries have potential, and their herbal research are developing. It is known that India also has a lot of studies focused on herbs and depigmentation (Mukherjee et al., 2018). Further, we grouped studies based on reagent types. Unsurprisingly, studies mainly focused on herbal extracts (38 studies, 42.2%) and active ingredients (38 studies, 42.2%), and they share the same proportion. Besides, there are 13 studies reported both extracts and active ingredients (14.4%, labeled “Combined”), but only 1 study involved herbal formulas (**Figure 1B**). The result may have something to do with the difficulties in studying three types of herbal reagents. As mentioned above, there are too many distractions in studying formulas.

The whitening market is still growing in Asia, research advances and market trends complement each other. According to our data, from 2017 to 2018, the number of studies increased from 25 to 35; but curiously, the number fell to 23 in 2019 and was not getting better in the first half of 2020 (**Figure 1C**). However, on the one hand, this could be a mistake caused by article collection, because we only reviewed the 300 most cited articles; on the other hand, we can’t rule out the impacts of delay in database updating of Web of Science. Likewise, studies in Korea was increased in 2018 and decreased in 2019, but China was still on the rise (**Figure 1D**). Partly because China’s scientific research, as a rising star, is developing fast. Though herbal research has not met a bottleneck, we should be aware that we already face some challenges. With the explosion of studies in skin whitening, the requirements are getting higher now.

Then, we further subdivided the studies by three reagent types and analyzed the differences between years and countries. Though the overall attention paid to herbal extracts and active ingredients is almost the same (**Figure 1B**), it seems like the proportion of extract-related studies was increasing from 2017 to 2019, so did that of combined studies (reported both extracts and active ingredients). In contrast, the proportion of ingredient-related studies had a clear decline (**Figure 1E**). We don’t yet know what this change means but have speculation. As new techniques and research facilities become more readily available, researchers may prefer to screen raw herbal materials rather than purchase purified ingredients, this helps to discover new reagents. Moreover, the attention paid to herbal extracts and active ingredients is slightly different between Korea and China, while Thailand and Japan have too few studies to be representative (**Figure 1F**).

### Quality and Value of Asian Herbal Studies

There is no doubt that Asian herbal research is developing well in skin whitening, and plenty of effective reagents have been found. But sadly, scattered studies are easy to ignore, their findings may also have limitations. Thus, to learn more about the overall quality of Asian herbal studies, we moved on and evaluated the scientificity and academic value of 90 collected studies.

**Figure 2:**
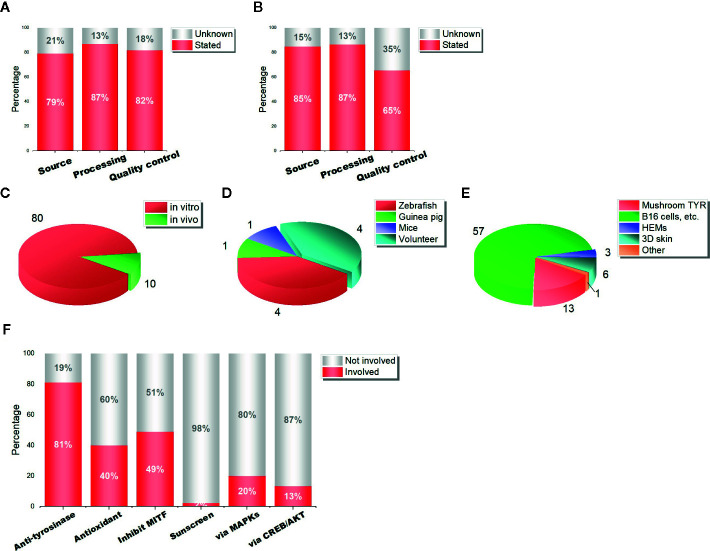
Scientificity and academic value of 90 Asian herbal studies. **(A)** the quality control of ingredient-related studies; **(B)** the quality control of mixture-related studies (herbal formula, extracts, and combined studies); **(C)** the number of *in vitro* and *in vivo* studies; **(D)** the models of *in vivo* studies; **(E)** the models of *in vitro* studies; **(F)** the percentage of different mechanisms involved in studies.

First, we divided studies into two groups: active ingredients and mixtures (including herbal extracts, formula, and combined studies). The evaluation criteria include information about (1) the source of herbal materials and ingredients (Source), (2) the processing method of raw materials (Processing), (3) the composition determination method of herbal extracts and ingredients, such as HPLC and UPLC (Quality control). For purchased herbal reagents, we assumed they met three criteria if detailed merchant information was provided. In this way, about 80% of the studies stated the sources and processing methods of herbal materials (**Figures 2A, B**). But only 65% of mixture-related studies provided quality control information (**Figure 2B**), which means that nearly 1/3 of the studies can not guarantee the quality of herbal extracts. Moreover, 82% of ingredient-related studies performed quality control (**Figure 2A**), but that does not mean they are scientific enough, because few of them stated the purity of the compounds. Therefore, we should be cautious about the credibility of these studies.

To assess the academic value, we also calculated the research models in 90 studies. It is surprised to find that only 10 (11.1%) of them carried out *in vivo* experiments (**Figure 2C**), the models include volunteers, zebrafish, mice, and guinea pigs (**Figure 2D**). The other 80 studies conducted *in vitro* experiments (**Figure 2E**), 57 (71.3%) of them used only mushroom tyrosinase, 13 (16.3%) of them used only mice cell lines (mostly B16 cells) and human melanoma cell lines, with or without mushroom tyrosinase; only 9 studies used human epidermal melanocytes (HEMs) and 3-dimensional human skin equivalents (3D skin). That is to say, only 1/10 of the studies showed the *in vivo* effects of herbs, and another 1/10 showed whitening evidence in human melanocytes. Although this is an acceptable proportion, there is room for further improvement.

**Figure 3:**
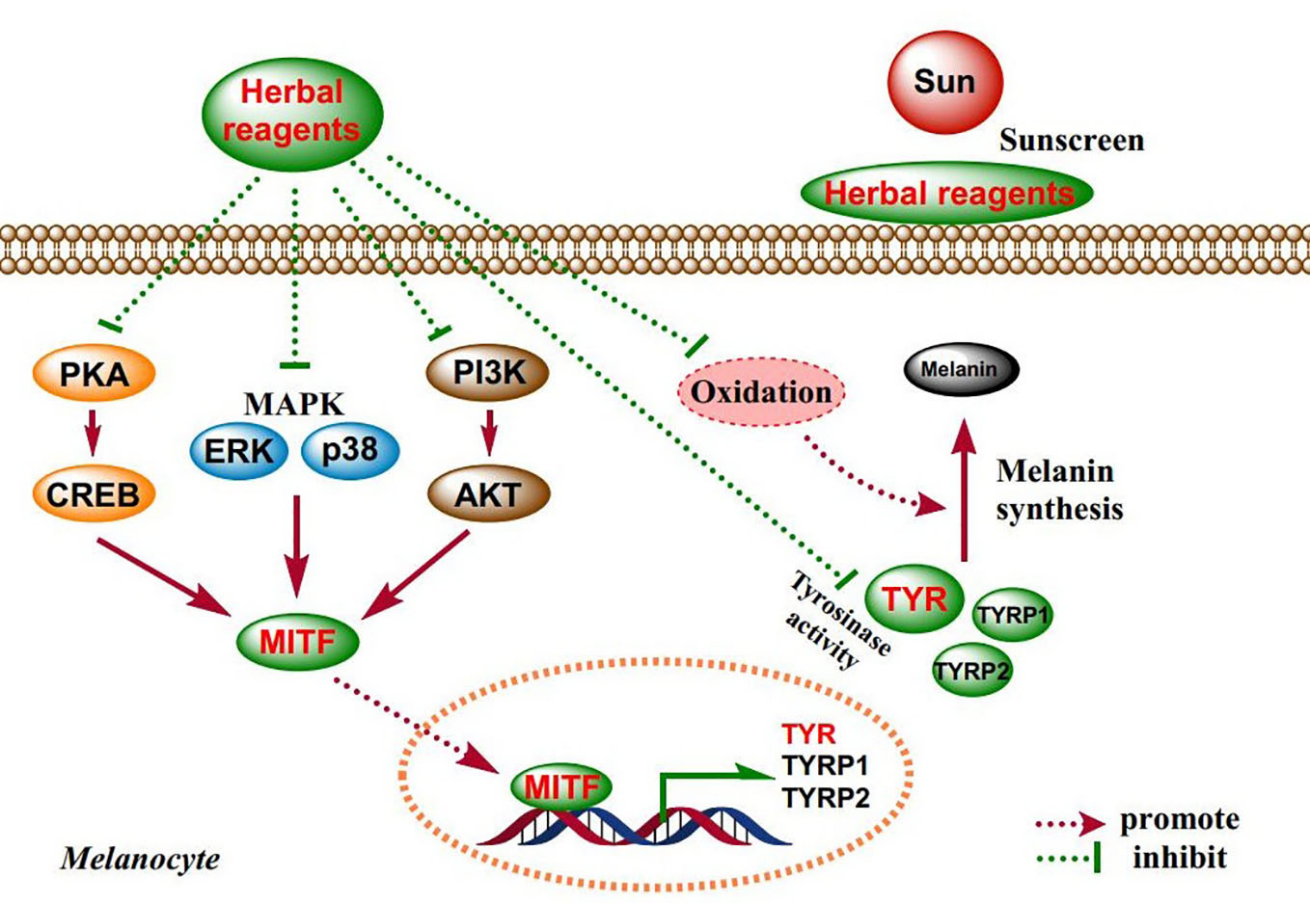
Schematic diagram of the mechanisms of herbal reagents in 90 studies.

In the 90 studies we collected, most herbal reagents regulate melanogenesis *via* part of the mechanisms previously mentioned. About 81% of the studies reported a decrease in tyrosinase activity, 40% reported antioxidant effect, and 49% observed downregulation of MITF expression. Otherwise, about 2% of the studies explored the sunscreen effect. When it comes to further mechanisms, only 20% of the studies reported a change in MAPK signaling pathway (mostly ERK and p38), and 13% involved CREB/AKT signaling pathway, etc. (**Figure 2F**). The main mechanisms of these herbal reagents are showed in **Figure 3**. Besides, the data show that studies are mainly focused on the ani-tyrosinase and antioxidant effects of herbal reagents, which are common characteristics of most herbs, thus their biological effects have not been thoroughly explored.

## Challenges and Prospects

In Asia, whitening cosmetics are in high demand, and leading to great progress in herbal research. However, the downside also deserves attention. In this work, we reviewed whitening-related studies from a comprehensive view and found some problems. One of them is that herbal components are complex and easily affected by multiple factors, which brings problems to studying herbal formula and extract, and also affects the reliability of the results. In contrast, active ingredients are the focus of research. The quality of most studies is reliable, but ingredient purification is a tough job. In a big way, Asian herbal research is not deep and systematic enough, because studies still focus on the discovery of new ingredients, rather than mechanism exploration and application. Also, there are few clinical studies on whitening. At present, the market for natural whitening cosmetics is a mess. On the one hand, research findings rarely translate into products; on the other hand, substandard products emerge one after another due to supervision loopholes (Desmedt et al., 2016). Many herbal reagents exhibit pharmacological properties (Zeng et al., 2019a) and can affect the structure and function of cells and organs, including the skin (Imokawa, 2008). Although herbal reagents are moderate, their side effects should be taken seriously, which have been ignored in the past.

In order to promote the application and development of traditional Asian herbs in whitening, all issues should be addressed carefully. Such as, the focus of herbal research should shift from quantity to quality and promote achievements transformation. At present, researchers are trying to synthesize herbal ingredients and derivatives, aiming to enhance their advantages and improve defects (Gonzalez-Sabin et al., 2011). From another point of view, synthetic and semi-synthetic natural ingredients may become a new hotspot in the future (Lee et al., 2016; Pillaiyar et al., 2017). In Asia, traditional herbs have been used over thousands of years, but have been ignored in the past century. In skin whitening, the discovery of natural ingredients such as arbutin was a surprise (Akiu et al., 1991; Chakraborty et al., 1998). In China alone, more than 10,000 herbs have been recorded (Xie and Yu, 1996), compared with this huge “herbal ingredient pool,” the findings so far have only scratched the surface (Zhao et al., 2020). Given the advances of techniques and the explosion of research, traditional Asian herbs need a new stage towards application.

## Search Strategies

Most materials in this review were obtained from Web of Science and PubMed; only documents covered by Science Citation Index (SCI) were selected. A few materials were obtained from ancient medical books, which were collected in the library of Central South University. Some keywords used in retrieving are provided below: Asian herbs, traditional herbs, melanogenesis, skin whitening. The 90 studies collected for statistical purposes were not directly cited in the paper, and their information were provided in the **Supplementary Materials**.

## Author Contributions

YH: collected the materials and drafted the manuscript. HZ: participated in the collection and screening of studies and provided analytical methods. JH: guided the analysis and arrangement of literature. LJ: recorded information and made tables. QZ and JC: put forward ideas, provided a framework for writing, checked and corrected writing problems. All authors contributed to the article and approved the submitted version.

## Funding

The authors thank the financial support from the National Natural Science Foundation of China (No. 81703101), the New Xiangya Talent Projects of the Third Xiangya Hospital of Central South University (No. JY201623 and No. 20170301), the Natural Science Foundation of Hunan Province (No. 2018JJ3788 and No. 2018JJ3793), the Project of Health and Family Planning Commission of Hunan Province (No. C2019173), and the Fundamental Research Funds for the Central Universities of Central South University (No. 2020zzts199).

## Conflict of Interest

The authors declare that the research was conducted in the absence of any commercial or financial relationships that could be construed as a potential conflict of interest.
