# Supplementary material for: Traditional Asian Herbs in Skin Whitening: The Current Development and Limitations
Source: Front Pharmacol. 2020 Jul 7;11:982. doi: 10.3389/fphar.2020.00982 (PMC7358643; doi:10.3389/fphar.2020.00982)
Supplement: Supplementary file 1 [file DataSheet_1.docx]

Supplementary Material

# Table S1. The information of Asian herbal formulas in this review

| **Study** | **Formula** | **Source** | **Species, concentration** | **Quality control reported? (Y/N)** | **Chemical analysis reported? (Y/N)** |
| --- | --- | --- | --- | --- | --- |
| Ye et al.  (2010a);  Tsang et al.  (2012) | Qian-wang-hong-bai-san | Purchased from retail drugstores in Shanghai, authenticated by Chinese Materia Medica, Shanghai University of Traditional Chinese Medicine, China, prepared by Ye et al. (2010) | tubers of *Bletilla striata* (Thunb.)Reichb. f.,  tubers of *Typhonium giganteum*Engl.,  pericarps of*Punica granatum* L.,  fruits of *Benincasa hispida* (Thunb.) Cogn.,  With the ratio of1:1:1:1. | Y - Prepared according to Pharmacopoeia ofthePeople’s Republic ofChina | Y - HPLC |
| Ye et al.  (2010) | Qiong-yu-gao | Purchased from retail drugstores in Shanghai, authenticated by Chinese Materia Medica, Shanghai University of Traditional Chinese Medicine, China, prepared by Ye et al. (2010) | roots and rhizomes of *Panax ginseng* C. A. Mey.,  roots of *Rehmannia glutinosa* Libosch.,  rhizomes of *Smilax glabra*Roxb.  With the ratio of 1:1:1. | Y - Prepared according to Pharmacopoeia ofthePeople’s Republic ofChina | Y - HPLC |
| Ye et al.  (2010a);  Ye et al.  (2010b) | San-bai-tang | Purchased from retail drugstores in Shanghai, authenticated by Chinese Materia Medica, Shanghai University of Traditional Chinese Medicine, China, prepared by Ye et al. (2010) | rhizomes of *Atractylodes macrocephala* Koidz.,  rhizomes of *Smilax glabra* Roxb.,  roots of *Paeonia lactiflora* Pall.,  With the ratio of1:1:1. | Y - Prepared according to Pharmacopoeia ofthePeople’s Republic ofChina | Y - HPLC |
| Panich etal.  (2013) | Ayurved Siriraj Brand Wattana formula (AVS073) | Obtained from Manufacturing Unit of Herbal Medicines and Products, manufactured byAyurved Thamrong School, Center of Applied Thai Traditional Medicine (CATTM), Faculty of Medicine Siriraj Hospital, Mahidol University, Thailand. | *Piper nigrum* (L.),  *Boesenbergia rotunda* (L.) Manf.,  *Cyperus rotundus* (L.),  ***Tinospora crispa****,  *Terminalia chebula* Retz.,  ***Cladogynos orientalis****,  *Derris scandens* (Roxb.) Benth.,  ***Anamirta cocculus* L.***,  ***Drypetes roxburghii* (Wall.)***,  *Cinnamomum siamense* Craib.,  *Ferulaa assa-foetida* L.,  ***Aegle maemelos* L.***,  *Conioselinum univittatum* Trucz.,  ***Saussurea lappa* Clark.***,  *Cryptolepis buchananii* Roem. & Schult.  Concentration not provided. | 1. Prepared according to GMP, Thailand. | Y - TLC & UHPLC |
| Kim et al.  (2016) | LASAP-C | Certified by Korea Food and Drug Administrationand purchased from a local herbal market in South Korea. A voucher specimen has been deposited at the Herbarium of the College of Korean Medicine, Dong-guk University, Ilsan, Korea, prepared by Kim et al. (2016) | root of ***Rehmannia glutinosa* Libosch. var. *purpurea* Makino***, 100g  fruit of *Lycium chinense* Mill., 50g  root of *Scutellaria baicalensis* Georgi, 50g  root of *Angelica dahurica* Bentham et Hooker f., 35g | Y - LASAP-C was extracted with 1 L distilled water at 100 °C for 4 h by using a Soxhlet extractor. The extract was filtered through a filter paper, and the filtrate was freeze-dried (yield, 62 g) and maintained at 4 °C. | Y - UPLC |
| Biswas et al.  (2016) | Ubtan | The rhizome of Curcuma longa L. (family - Zingiberaceae), seeds of Cicer arietinum L. (family - Fabaceae) and heartwood of Santalum album L. (family - Santalaceae) were purchased from an authorized Ayurvedic herb vendor, Jadavpur, Kolkata, India in the month of October, 2012. Plant specimens were identified and authenticated by Dr. S. Rajan, Field botanist, the medicinal plant collection unit, Ooty, Tamilnadu, Govt. of India. | rhizome of *Curcuma longa L.*  seeds of *Cicer arietinum L.*  heartwood of *Santalum album L.* | Y - Evaluation of physicochemical properties of the optimized formulation (UF-1) has been carried out by analysis of pH, flow properties and stability. | Y - RP-HPLC and HPTLC |

** The name used is not consistent with full botanical taxonomic name as validated by Kewscience (http://mpns.kew.org/mpns-portal) or The Plant List (www.theplantlist.org).*

# Table S2. The information of herbal extracts included in this review

| **Study** | **species, source, concentration** | **Quality control reported? (Y/N)** | **Chemical analysis reported? (Y/N)** |
| --- | --- | --- | --- |
| Jimenez-Perez et al., 2018 | Dry leaves of ***Panax ginseng****  [Gochang, Korea] | Y - 15 g leaves were shade-dried for 6-8 h, grounded and boiled for 20 min in 100 ml of sterile water, the collected extract was filtered and centrifuged at 10,000 rpm for 10 min, the total volume of filtrate was maintained at 100 ml. | N |
| Huang et al., 2014 | Roots of *Lycium chinense* Mill.  [Guanyin, Taiwan, identified] | Y - The roots were sliced into pieces and exposed to sunlight for 7 more days and then dried at 80°C for 2 h in an oven, then pulverized to a fine powder (#20 mesh), extracted by SFE. | Y - HPLC |
| Goh et al., 2018 | ***Polypodium leucotomos****  [Fernblock®, IFC, Madrid, Spain] | Y - Marketed drug | N - Marketed drug |
| Shim et al., 2017 | *Gastrodia elata* Blume [Chuncheon, Korea] | Y - GEB ground, powder (10 g) was extracted in 200 mL of de-ionized H2O at 90°C for 3 h, centrifugation at 4,500 rpm. The supernatant was filtered using filter paper with 6 μm pore size and concentrated under vacuum. | N |
| Nam and Lee, 2016 | fruits of***Foeniculum vulgare****  [Gyeongju, Korea, identified] | Y - Air-dried and powdered plant materials (500 g) were extracted with hot methanol for 3 h to obtain crude extract, then partitioned with n-hexane, methylene chloride, ethyl acetate, and n-butanol to afford the corresponding fractions. The hexane fraction was subjected to silica gel column chromatographic separation to yield subfractions. | Y - HPLC |
| Wang et al., 2014 | Seeds of ***Cuscuta chinensis****  [Guangzhou, China, authenticated] | Y - Dry seeds (30 g) were immersed in 500 mL of distilled water for 8 h and boiled under reflux for 90 min, twice. The resultant extracts were merged, centrifuged, the filtrate was evaporated to a paste, next diluted with distilled water to 0.3 g/mL. Or extracted with 95% ethanol under reflux twice, and diluted with distilled water to 0.3 g/mL. | Y - HPLC |
| Hu et al., 2019 | *Ganoderma lucidum*  [Xi’an, China, purchased] | Y - Marketed drug | N - Marketed drug |
| Jiang et al., 2019 | *Ganoderma lucidum*  [Xi’an, China, purchased] | Y - Marketed drug | N - Marketed drug |
| Suganya et al., 2015 | *Pogostemon plectrantoides Desf.*  [Western Ghats, Central India and Northeast India] | Y - The shade-dried leaves were subjected to hydro- distillation in a Clevenger apparatus for about 8 h. The species from all three regions yielded dark brown color volatile oils with strong woody odour.The EOs were dried over anhydrous sodium sulphate and stored at 4 ◦C. | Y - GC-MS |

** The name used is not consistent with full botanical taxonomic name as validated by Kewscience (http://mpns.kew.org/mpns-portal) or The Plant List (www.theplantlist.org).*

# Table S3. The information of active herbal ingredients included in this review

| **Study** | **Compound, concentration** | **Source** | **Purity (%)** | **Quality control reported (Y/N)** |
| --- | --- | --- | --- | --- |
| Chen et al., 2016 | glabridin | Push Bio-technology (Chengdu,China) | 98.5% | Y |
| Lee et al., 2017 | floralginsenoside A | Purified by Lee et al., 2017 | Not provided | Y |
| Ali et al., 2012 | aloin  yohimbine  reserpine | Sigma Chemical Co. | 99 %  98 %  99 % | Y - HPLC |
| Ho et al., 2016 | Lutein  carotenoids | Fluka Chemical Co. (Buchs,Switzerland)  Purified by Ho et al., 2016 | 91.5–99.9% | Y - HPLC |
| Manda et al., 2014 | mitragynine | Purified by Ali et al., 2013 | ≥95% | Y |
| [Biswas et al., 2017](#_ENREF_7) | betulinic acid | Procured (Jadavpur, Kolkata, India )  Authenticated and identified by a field botanist | 97.14% | Y - HPLC |
| Anantharaman et al., 2016 | Bixin and norbixin | N | Ultraviolet visible spectroscopy / FTIR spectra | Y - HPLC |
| [Kundu and Mitra, 2014](#_ENREF_33) | 2-hydroxy-4-mehoxybenzaldehyde (MBALD) | Indian Institute of Technology Kharagpur campus, India  identified by Pranjit Sarma | UV–vis spectral analysis / GC-MS | Y - HPLC |
