# Supplementary material for: Traditional Asian Herbs in Skin Whitening: The Current Development and Limitations
Source: Front Pharmacol. 2020 Jul 7;11:982. doi: 10.3389/fphar.2020.00982 (PMC7358643; doi:10.3389/fphar.2020.00982)
Supplement: Supplementary file 2 [file DataSheet_2.docx]

The list of 90 studies

Ahn, H.-Y., Choo, Y.-M., and Cho, Y.-S. (2018). Anti-Pigmentation Effects of Eight Phellinus linteus-Fermented Traditional Crude Herbal Extracts on Brown Guinea Pigs of Ultraviolet B-Induced Hyperpigmentation. Journal of Microbiology and Biotechnology 28, 375-380.

Allam, A.E., Nafady, A.M., Nakagawa, T., Takemoto, N., and Shimizu, K. (2018). Effect of polyphenols from Vicia faba L on lipase activity and melanogenesis. Natural Product Research 32, 1920-1925.

Amen, Y., Arung, E.T., Afifi, M.S., Halim, A.F., Ashour, A., Fujimoto, R., Goto, T., and Shimizu, K. (2017). Melanogenesis inhibitors from Coix lacryma-jobi seeds in B16-F10 melanoma cells. Natural Product Research 31, 2712-2718.

Arung, E.T., Sinamabela, J.R., Rosamah, E., Kusuma, I.W., Kuspradini, H., Alam, A.E., Amen, Y., Tanaka, H., Satria, D., Shimizu, K., and Ishikawa, H. (2019). Antioxidant and Antimelanogenesis Activities of Glyasperin A From Macaranga pruinosa Leaves. Natural Product Communications 14.

Bae, I.-H., Lee, E.S., Yoo, J.W., Lee, S.H., Ko, J.Y., Kim, Y.J., Lee, T.R., Kim, D.-Y., and Lee, C.S. (2019). Mannosylerythritol lipids inhibit melanogenesis via suppressing ERK-CREB-MiTF-tyrosinase signalling in normal human melanocytes and a three-dimensional human skin equivalent. Experimental Dermatology 28, 738-741.

Byun, E.-B., Song, H.-Y., Mushtaq, S., Kim, H.-M., Kang, J.A., Yang, M.-S., Sung, N.-Y., Jang, B.-S., and Byun, E.-H. (2017). Gamma-Irradiated Luteolin Inhibits 3-Isobutyl-1-Methylxanthine-Induced Melanogenesis Through the Regulation of CREB/MITF, PI3K/Akt, and ERK Pathways in B16BL6 Melanoma Cells. Journal of Medicinal Food 20, 812-819.

Chae, J.K., Subedi, L., Jeong, M., Park, Y.U., Kim, C.Y., Kim, H., and Kim, S.Y. (2017). Gomisin N Inhibits Melanogenesis through Regulating the PI3K/Akt and MAPK/ERK Signaling Pathways in Melanocytes. International Journal of Molecular Sciences 18.

Chatatikun, M., and Chiabchalard, A. (2017). Thai plants with high antioxidant levels, free radical scavenging activity, anti-tyrosinase and anti-collagenase activity. Bmc Complementary and Alternative Medicine 17.

Chatatikun, M., Yamauchi, T., Yamasaki, K., Aiba, S., and Chiabchalard, A. (2019). Anti melanogenic effect of Croton roxburghii and Croton sublyratus leaves in alpha-MSH stimulated B16F10 cells. Journal of traditional and complementary medicine 9, 66-72.

Cheng, M.-C., Lee, T.-H., Chu, Y.-T., Syu, L.-L., Hsu, S.-J., Cheng, C.-H., Wu, J., and Lee, C.-K. (2018). Melanogenesis Inhibitors from the Rhizoma of Ligusticum Sinense in B16-F10 Melanoma Cells In Vitro and Zebrafish In Vivo. International Journal of Molecular Sciences 19.

Cho, H., Kim, O., Lee, Y., Kang, L.-J., Nguyen, C.N., Ishihara, A., and Kim, H.-E. (2017). Feruloylserotonin inhibits hydrogen peroxide-induced melanogenesis and apoptosis in B16F10 and SK-Mel-2 melanoma cells. Biochemical and Biophysical Research Communications 491, 973-979.

Cho, U.M., Choi, D.H., Yoo, D.S., Park, S.J., and Hwang, H.S. (2019). Inhibitory Effect of Ficin Derived from Fig Latex on Inflammation and Melanin Production in Skin Cells. Biotechnology and Bioprocess Engineering 24, 288-297.

Choodej, S., Pudhom, K., Yamauchi, K., and Mitsunaga, T. (2019). Inhibition of melanin production by sesquiterpene lactones from Saussurea lappa and their analogues. Medicinal Chemistry Research 28, 857-862.

Chung, K.W., Jeong, H.O., Lee, E.K., Kim, S.J., Chun, P., Chung, H.Y., and Moon, H.R. (2018a). Evaluation of Antimelanogenic Activity and Mechanism of Galangin in Silico and in Vivo. Biological & Pharmaceutical Bulletin 41, 73-79.

Chung, Y.C., Ko, J.-H., Kang, H.-K., Kim, S., Kang, C.I., Lee, J.N., Park, S.-M., and Hyun, C.-G. (2018b). Antimelanogenic Effects of Polygonum tinctorium Flower Extract from Traditional Jeju Fermentation via Upregulation of Extracellular Signal-Regulated Kinase and Protein Kinase B Activation. International Journal of Molecular Sciences 19.

Chunhakant, S., and Chaicharoenpong, C. (2019). Antityrosinase, Antioxidant, and Cytotoxic Activities of Phytochemical Constituents from Manilkara zapota L. Bark. Molecules 24.

Dai, C.-Y., Liu, P.-F., Liao, P.-R., Qu, Y., Wang, C.-X., Yang, Y., and Cui, X.-M. (2018). Optimization of Flavonoids Extraction Process in Panax notoginseng Stem Leaf and a Study of Antioxidant Activity and Its Effects on Mouse Melanoma B16 Cells. Molecules 23.

Eghbah-Feriz, S., Taleghani, A., Al-Najjar, H., Emami, S.A., Rahimi, H., Asili, J., Hasanzadeh, S., and Tayarani-Najaran, Z. (2018). Anti-melanogenesis and anti-tyrosinase properties of Pistacia atlantica subsp. mutica extracts on B16F10 murine melanoma cells. Research in Pharmaceutical Sciences 13, 533-545.

Farvin, K.H.S., Surendraraj, A., Al-Ghunaim, A., and Al-Yamani, F. (2019). Chemical profile and antioxidant activities of 26 selected species of seaweeds from Kuwait coast. Journal of Applied Phycology 31, 2653-2668.

Fernando, I.P.S., Sanjeewa, K.K.A., Samarakoon, K.W., Kim, H.-S., Gunasekara, U.K.D.S.S., Park, Y.-J., Abeytunga, D.T.U., Lee, W.W., and Jeon, Y.-J. (2018). The potential of fucoidans from Chnoospora minima and Sargassum polycystum in cosmetics: antioxidant, anti-inflammatory, skin-whitening, and antiwrinkle activities. Journal of Applied Phycology 30, 3223-3232.

Fukunaga, S., Wada, S., Aoi, W., Osada-Oka, M., Minamiyama, Y., Ichikawa, H., and Higashi, A. (2018). Effect of melanogenesis inhibition by a yeast extract in comparison to that by other food extracts, and its mechanism of action. Journal of Food Biochemistry 42.

Guo, L., Yin, Z., Wen, L., Xin, J., Gao, X., and Zheng, X. (2019). Flower extracts from Paeonia decomposita and Paeonia ostii inhibit melanin synthesis via cAMP-CREB-associated melanogenesis signaling pathways in murine B16 melanoma cells. Journal of Food Biochemistry 43.

Han, H.J., Park, S.K., Kang, J.Y., Kim, J.M., Yoo, S.K., and Heo, H.J. (2020). Anti-Melanogenic Effect of Ethanolic Extract of Sorghum bicolor on IBMX-Induced Melanogenesis in B16/F10 Melanoma Cells. Nutrients 12.

Hashemi-Shahri, S.H., Golshan, A., Mohajeri, S.A., Baharara, J., Amini, E., Salek, F., Sahebkar, A., and Tayarani-Najaran, Z. (2018). ROS-scavenging and Anti-tyrosinase Properties of Crocetin on B16F10 Murine Melanoma Cells. Anti-Cancer Agents in Medicinal Chemistry 18, 1064-1069.

Ho, Y.-S., Wu, J.-Y., and Chang, C.-Y. (2019). A New Natural Antioxidant Biomaterial from Cinnamomum osmophloeum Kanehira Leaves Represses Melanogenesis and Protects against DNA Damage. Antioxidants 8.

Homma, T., Kageyama, S., Nishikawa, A., and Nagata, K. (2020). Anti-melanogenic activity of salacinol by inhibition of tyrosinase oligosaccharide processing. Journal of biochemistry 167, 503-511.

Hu, S., Zheng, Z., Chen, F., and Wang, M. (2017). The depigmenting effect of natural resorcinol type polyphenols Kuwanon O and Sanggenon T from the roots of morus australis. Journal of Ethnopharmacology 195, 196-203.

Im, K.H., Baek, S.A., Choi, J., and Lee, T.S. (2019). Antioxidant, Anti-Melanogenic and Anti-Wrinkle Effects of Phellinus vaninii. Mycobiology 47, 494-505.

Jesumani, V., Du, H., Pei, P., Zheng, C., Cheong, K.-L., and Huang, N. (2019). Unravelling property of polysaccharides from Sargassum sp. as an anti-wrinkle and skin whitening property. International Journal of Biological Macromolecules 140, 216-224.

Jin, Y., Kim, J.H., Hong, H.-D., Kwon, J., Lee, E.J., Jang, M., Lee, S.-Y., Han, A.-R., Nam, T.G., Hong, S.K., Huh, T.-L., Kang, N.J., and Lim, T.-G. (2018). Ginsenosides Rg5 and Rkl, the skin-whitening agents in black ginseng. Journal of Functional Foods 45, 67-74.

Jiratchayamaethasakul, C., Ding, Y., Hwang, O., Im, S.-T., Jang, Y., Myung, S.-W., Lee, J.M., Kim, H.-S., Ko, S.-C., and Lee, S.-H. (2020). In vitro screening of elastase, collagenase, hyaluronidase, and tyrosinase inhibitory and antioxidant activities of 22 halophyte plant extracts for novel cosmeceuticals. Fisheries and Aquatic Sciences 23, 6-Article No.: 6.

Kang, S.-H., Jeon, Y.-D., Cha, J.-Y., Hwang, S.-W., Lee, H.-Y., Park, M., Lee, B.-R., Shin, M.-K., Kim, S.-J., Shin, S.-M., Kim, D.-K., Jin, J.-S., and Lee, Y.-M. (2018). Antioxidant and skin-whitening effects of aerial part of Euphorbia supina Raf. Extract. Bmc Complementary and Alternative Medicine 18.

Kim, D.-Y., Won, K.-J., Hwang, D.I., Park, S.M., Kim, B., and Lee, H.M. (2018a). Chemical Composition, Antioxidant and Anti-melanogenic Activities of Essential Oils from Chrysanthemum boreale Makino at Different Harvesting Stages. Chemistry & Biodiversity 15.

Kim, J.H., Cho, I.S., So, Y.K., Kim, H.-H., and Kim, Y.H. (2018b). Kushenol A and 8-prenylkaempferol, tyrosinase inhibitors, derived from Sophora flavescens. Journal of Enzyme Inhibition and Medicinal Chemistry 33, 1048-1054.

Kim, J.H., Lee, S., Park, S., Park, J.S., Kim, Y.H., and Yang, S.Y. (2019). Slow-Binding Inhibition of Tyrosinase by Ecklonia cava Phlorotannins. Marine Drugs 17.

Kim, J.H., Yun, E.J., Yu, S., Kim, K.H., and Kang, N.J. (2017a). Different Levels of Skin Whitening Activity among 3,6-Anhydro-l-galactose, Agarooligosaccharides, and Neoagarooligosaccharides. Marine Drugs 15.

Kim, S.E., Lee, C.M., and Kim, Y.C. (2017b). Anti-Melanogenic Effect of Oenothera laciniata Methanol Extract in Melan-a Cells. Toxicological Research 33, 55-62.

Kim, Y., Lee, S., Ryu, J.H., Yoon, K.D., and Shin, S.S. (2018c). Effect of Aurea Helianthus stem extract on anti-melanogenesis. Bioscience Biotechnology and Biochemistry 82, 1871-1879.

Ko, G.-A., and Cho, S.K. (2018). Phytol suppresses melanogenesis through proteasomal degradation of MITF via the ROS-ERK signaling pathway. Chemico-Biological Interactions 286, 132-140.

Ko, G.-A., Shrestha, S., and Cho, S.K. (2018). Sageretia thea fruit extracts rich in methyl linoleate and methyl linolenate downregulate melanogenesis via the Akt/GSK3 beta signaling pathway. Nutrition Research and Practice 12, 3-12.

Kudo, M., Kobayashi-Nakamura, K., and Tsuji-Naito, K. (2017). Bifunctional effects of O-methylated flavones from Scutellaria baicalensis Georgi on melanocytes: Inhibition of melanin production and intracellular melanosome transport. Plos One 12.

Lee, B., Moon, K.M., Lee, B.-S., Yang, J.-H., Park, K.I., Cho, W.-K., and Ma, J.Y. (2017a). Swertiajaponin inhibits skin pigmentation by dual mechanisms to suppress tyrosinase. Oncotarget 8, 95530-95541.

Lee, C.-S., Nam, G., Bae, I.-H., and Park, J. (2019a). Whitening efficacy of ginsenoside F1 through inhibition of melanin transfer in cocultured human melanocytes-keratinocytes and three-dimensional human skin equivalent. Journal of Ginseng Research 43, 300-304.

Lee, C.S., Baek, H.S., Bae, I.H., Choi, S.J., Kim, Y.J., Lee, J.H., and Kim, J.W. (2018a). Depigmentation efficacy of galacturonic acid through tyrosinase regulation in B16 murine melanoma cells and a three-dimensional human skin equivalent. Clinical and Experimental Dermatology 43, 708-712.

Lee, D.Y., Kim, H.-G., Lee, Y.-G., Kim, J.H., Lee, J.W., Choi, B.-R., Jang, I.-B., Kim, G.-S., and Baek, N.-I. (2018b). Isolation and Quantification of Ginsenoside Rh23, a New Anti-Melanogenic Compound from the Leaves of Panax ginseng. Molecules 23.

Lee, H., and Cha, H.J. (2018). Poria cocos Wolf extracts represses pigmentation in vitro and in vivo. Cellular and Molecular Biology 64, 80-84.

Lee, H.J., Sim, M.O., Woo, K.W., Jeong, D.-E., Jung, H.K., An, B., and Cho, H.W. (2019b). Antioxidant and Antimelanogenic Activities of Compounds Isolated from the Aerial Parts of Achillea alpina L. Chemistry & Biodiversity 16.

Lee, J.-O., Kim, E., Kim, J.H., Hong, Y.H., Kim, H.G., Jeong, D., Kim, J., Kim, S.H., Park, C., Seo, D.B., Son, Y.-J., Han, S.Y., and Cho, J.Y. (2018c). Antimelanogenesis and skin-protective activities of Panax ginseng calyx ethanol extract. Journal of Ginseng Research 42, 389-399.

Lee, J., Ryu, H.S., Kim, J.-M., Jung, T.-H., Park, S.-M., and Lee, Y.-M. (2017b). Anti-melanogenic effect of gomisin N from Schisandra chinensis (Turcz.) Baillon (Schisandraceae) in melanoma cells. Archives of Pharmacal Research 40, 807-817.

Lee, J.Y., Cho, Y.-R., Park, J.H., Ahn, E.-K., Jeong, W., Shin, H.S., Kim, M.-S., Yang, S.H., and Oh, J.S. (2019c). Anti-melanogenic and anti-oxidant activities of ethanol extract of Kummerowia striata: Kummerowia striata regulate anti-melanogenic activity through down-regulation of TRP-1, TRP-2 and MITF expression. Toxicology Reports 6, 10-17.

Lee, K.W., Ryu, H.W., Oh, S.-S., Park, S., Madhi, H., Yoo, J., Park, K.-H., and Kim, K.D. (2017c). Depigmentation of alpha-melanocyte-stimulating hormone-treated melanoma cells by beta-mangostin is mediated by selective autophagy. Experimental Dermatology 26, 585-591.

Lee, S.-W., Lim, J.-M., Mohan, H., Seralathan, K.-K., Park, Y.-J., Lee, J.-H., and Oh, B.-T. (2020). Enhanced bioactivity of Zanthoxylum schinifolium fermented extract: Anti-inflammatory, anti-bacterial, and anti-melanogenic activity. Journal of bioscience and bioengineering 129, 638-645.

Lee, S., Lee, D.-H., Kim, J.-C., Um, B.H., Sung, S.H., Jeong, L.S., Kim, Y.K., and Kim, S.-N. (2017d). Pectolinarigenin, an aglycone of pectolinarin, has more potent inhibitory activities on melanogenesis than pectolinarin. Biochemical and Biophysical Research Communications 493, 765-772.

Li, M.-X., Bai, X., Ma, Y.-P., Zhang, H.-X., Nama, N., Pei, S.-J., and Du, Z.-Z. (2019). Cosmetic potentials of extracts and compounds from Zingiber cassumunar Roxb. rhizome. Industrial Crops and Products 141.

Lim, J.W., Ha, J.H., Jeong, Y.J., and Park, S.N. (2018). Anti-melanogenesis effect of dehydroglyasperin C through the downregulation of MITF via the reduction of intracellular cAMP and acceleration of ERK activation in B16F1 melanoma cells. Pharmacological Reports 70, 930-935.

Lin, D., Wang, S.-H., Song, T.-Y., Hsieh, C.-W., and Tsai, M.-S. (2019). Safety and efficacy of tyrosinase inhibition of Paeonia suffruticosa Andrews extracts on human melanoma cells. Journal of Cosmetic Dermatology 18, 1921-1929.

Lin, Y.-S., Chen, H.-J., Huang, J.-P., Lee, P.-C., Tsai, C.-R., Hsu, T.-F., and Huang, W.-Y. (2017). Kinetics of Tyrosinase Inhibitory Activity Using Vitis vinifera Leaf Extracts. Biomed Research International.

Liu, Z.-J., Wang, Y.-L., Li, Q.-L., and Yang, L. (2018). Improved antimelanogenesis and antioxidant effects of polysaccharide from Cuscuta chinensis Lam seeds after enzymatic hydrolysis. Brazilian Journal of Medical and Biological Research 51.

Lorz, L.R., Yoo, B.C., Kim, M.-Y., and Cho, J.Y. (2019). Anti-Wrinkling and Anti-Melanogenic Effect of Pradosia mutisii Methanol Extract. International Journal of Molecular Sciences 20.

Lv, J., Fu, Y., Cao, Y., Jiang, S., Yang, Y., Song, G., Yun, C., and Gao, R. (2020). Isoliquiritigenin inhibits melanogenesis, melanocyte dendricity and melanosome transport by regulating ERK-mediated MITF degradation. Experimental Dermatology 29, 149-157.

Meer, S., and Akhtar, N. (2018). Annona muricata extract containing pharmaceutical emulgels with and without penetration enhancer for depigmenting and antierythmic effects. Pakistan Journal of Pharmaceutical Sciences 31, 2683-2688.

Mohammad, I.S., Naveed, M., Ijaz, S., Shumzaid, M., Hassan, S., Muhammad, K.S., Rasool, F., Akhtar, N., Ishaq, H.M., and Khan, H.M.S. (2018). Phytocosmeceutical formulation development, characterization and its in-vivo investigations. Biomedicine & Pharmacotherapy 107, 806-817.

Nile, S.H., Nile, A., Liu, J., Kim, D.H., and Kai, G. (2019). Exploitation of apple pomace towards extraction of triterpenic acids, antioxidant potential, cytotoxic effects, and inhibition of clinically important enzymes. Food and Chemical Toxicology 131.

Numonov, S., Bobakulov, K., Numonova, M., Sharopov, F., Setzer, W.N., Khalilov, Q., Begmatov, N., Habasi, M., and Aisa, H.A. (2018). New coumarin from the roots of Prangos pabularia. Natural Product Research 32, 2325-2332.

Oh, T.-I., Jung, H.-J., Lee, Y.-M., Lee, S., Kim, G.-H., Kan, S.-Y., Kang, H., Oh, T., Ko, H.M., Kwak, K.-C., and Lim, J.-H. (2018). Zerumbone, a Tropical Ginger Sesquiterpene of Zingiber officinale Roscoe, Attenuates -MSH-Induced Melanogenesis in B16F10 Cells. International Journal of Molecular Sciences 19.

Oh, T.-I., Yun, J.-M., Park, E.-J., Kim, Y.-S., Lee, Y.-M., and Lim, J.-H. (2017). Plumbagin Suppresses -MSH-Induced Melanogenesis in B16F10 Mouse Melanoma Cells by Inhibiting Tyrosinase Activity. International Journal of Molecular Sciences 18.

Ozer, M.S., Kirkan, B., Sarikurkcu, C., Cengiz, M., Ceylan, O., Atilgan, N., and Tepe, B. (2018). Onosma heterophyllum: Phenolic composition, enzyme inhibitory and antioxidant activities. Industrial Crops and Products 111, 179-184.

Park, E.-H., Bae, W.-Y., Kim, J.-Y., Kim, K.-T., and Paik, H.-D. (2017). Antimelanogenic effects of Inula britannica flower petal extract fermented by Lactobacillus plantarum KCCM 11613P. Journal of Zhejiang University-Science B 18, 816-824.

Park, H.-J., Cho, J.-H., Hong, S.-H., Kim, D.-H., Jung, H.-Y., Kang, I.-K., and Cho, Y.-J. (2018). Whitening and anti-wrinkle activities of ferulic acid isolated from Tetragonia tetragonioides in B16F10 melanoma and CCD-986sk fibroblast cells. Journal of Natural Medicines 72, 127-135.

Phacharapiyangkul, N., Thirapanmethee, K., Sa-Ngiamsuntorn, K., Panich, U., Lee, C.-H., and Chomnawang, M.T. (2019). Effect of Sucrier Banana Peel Extracts on Inhibition of Melanogenesis through the ERK Signaling Pathway. International Journal of Medical Sciences 16, 602-606.

Pi, K., and Lee, K. (2017). Prunus mume extract exerts antioxidant activities and suppressive effect of melanogenesis under the stimulation by alpha-melanocyte stimulating hormone in B16-F10 melanoma cells. Bioscience Biotechnology and Biochemistry 81, 1883-1890.

Pratoomthai, B., Songtavisin, T., Gangnonngiw, W., and Wongprasert, K. (2018). In vitro inhibitory effect of sulfated galactans isolated from red alga Gracilaria fisheri on melanogenesis in B16F10 melanoma cells. Journal of Applied Phycology 30, 2611-2618.

Ratanasumarn, N., and Chitprasert, P. (2020). Cosmetic potential of lignin extracts from alkaline-treated sugarcane bagasse: Optimization of extraction conditions using response surface methodology. International journal of biological macromolecules 153, 138-145.

Ren, G., Xue, P., Sun, X., and Zhao, G. (2018). Determination of the volatile and polyphenol constituents and the antimicrobial, antioxidant, and tyrosinase inhibitory activities of the bioactive compounds from the by-product of Rosa rugosa Thunb. var. plena Regal tea. Bmc Complementary and Alternative Medicine 18.

Sahin, S.C. (2018). The potential of Arthrospira platensis extract as a tyrosinase inhibitor for pharmaceutical or cosmetic applications. South African Journal of Botany 119, 236-243.

Shim, E., Song, E., Choi, K.S., Choi, H.-J., and Hwang, J. (2017). Inhibitory effect of Gastrodia elata Blume extract on alpha-melanocyte stimulating hormone-induced melanogenesis in murine B16F10 melanoma. Nutrition Research and Practice 11, 173-179.

Sim, M.-O., Ham, J.R., and Lee, M.-K. (2017). Young leaves of reed (Phragmites communis) suppress melanogenesis and oxidative stress in B16F10 melanoma cells. Biomedicine & Pharmacotherapy 93, 165-171.

Tepe, A.S., and Ozaslan, M. (2020). Anti-Alzheimer, anti-diabetic, skin-whitening, and antioxidant activities of the essential oil of Cinnamomum zeylanicum. Industrial Crops and Products 145.

Trivedi, M.K., Gangwar, M., Mondal, S.C., and Jana, S. (2017). Protective effects of tetrahydrocurcumin (THC) on fibroblast and melanoma cell lines in vitro: it's implication for wound healing. Journal of Food Science and Technology-Mysore 54, 1137-1145.

Wang, L.-X., Qian, J., Zhao, L.-N., and Zhao, S.-H. (2018). Effects of volatile oil from ginger on the murine B16 melanoma cells and its mechanism. Food & Function 9, 1058-1069.

Wang, S.-T., Chang, W.-C., Hsu, C., and Su, N.-W. (2017a). Antimelanogenic Effect of Urolithin A and Urolithin B, the Colonic Metabolites of Ellagic Acid, in B16 Melanoma Cells. Journal of Agricultural and Food Chemistry 65, 6870-6876.

Wang, Y.-C., Haung, X.-Y., Chiu, C.-C., Lin, M.-Y., Lin, W.-H., Chang, W.-T., Tseng, C.-C., and Wang, H.-M.D. (2019). Inhibitions of melanogenesis via Phyllanthus emblica fruit extract powder in B16F10 cells. Food Bioscience 28, 177-182.

Wang, Z.-J., Xu, W., Liang, J.-W., Wang, C.-S., and Kang, Y. (2017b). EFFECT OF FUCOIDAN ON B16 MURINE MELANOMA CELL MELANIN FORMATION AND APOPTOSIS. African journal of traditional, complementary, and alternative medicines : AJTCAM 14, 149-155.

Wuttisin, N., Boonmak, J., Thaipitak, V., Thitilertdecha, N., and Kittigowittana, K. (2017). Anti-tyrosinase activity of orange peel extract and cosmetic formulation. International Food Research Journal 24, 2128-2132.

Xie, X.-T., Zhang, X., Liu, Y., Chen, X.-Q., and Cheong, K.-L. (2020). Quantification of 3,6-anhydro-galactose in red seaweed polysaccharides and their potential skin-whitening activity. 3 Biotech 10.

Yang, S.H., Tsatsakis, A.M., Tzanakakis, G., Kim, H.-S., Le, B., Sifaki, M., Spandidos, D.A., Tsukamoto, C., Golokhvast, K.S., Izotov, B.N., and Chung, G. (2017). Soyasaponin Ag inhibits alpha-MSH-induced melanogenesis in B16F10 melanoma cells via the downregulation of TRP-2. International Journal of Molecular Medicine 40, 631-636.

Yoon, M.-Y., Kim, H.-J., and Lee, S.-J. (2019). The effect of antioxidant and whitening action on Plantago asiatica L. leaf ethanol extract for health care. Technology and Health Care 27, 567-577.

Yoshida, I., Ito, C., Matsuda, S., Tsuji, A., Yanaka, N., and Yuasa, K. (2017). Alisol B, a triterpene from Alismatis rhizoma (dried rhizome of Alisma orientale), inhibits melanin production in murine B16 melanoma cells. Bioscience Biotechnology and Biochemistry 81, 534-540.

Zhang, J., Wang, C., Wang, C., Sun, B., and Qi, C. (2018). Understanding the role of extracts from sea buckthorn seed residues in anti- melanogenesis properties on B16F10 melanoma cells. Food & Function 9, 5402-5416.

Zhao, Z., Ma, S., and Dong, X. (2019). Anti-melanogenesis Efficacy of Ginkgolide B is Favored by Attenuating Oxidative Stress and Melanin Synthesis in B16f1 Melanoma Cell Model. International Journal of Pharmacology 15, 560-566.
